# Dual Inhibiting Senescence and Epithelial-to-Mesenchymal Transition by Erythropoietin Preserve Tubular Epithelial Cell Regeneration and Ameliorate Renal Fibrosis in Unilateral Ureteral Obstruction

**DOI:** 10.1155/2013/308130

**Published:** 2013-11-19

**Authors:** Adis Tasanarong, Supranee Kongkham, Sookkasem Khositseth

**Affiliations:** ^1^Nephrology Unit, Department of Medicine, Faculty of Medicine, Thammasat University (Rangsit Campus), Khlong Nueng, Khlong Luang, Pathum Thani 12121, Thailand; ^2^Division of Biochemistry, Department of Preclinical Sciences, Faculty of Medicine, Thammasat University (Rangsit Campus), Khlong Nueng, Khlong Luang, Pathum Thani 12121, Thailand; ^3^Department of Paediatric, Faculty of Medicine, Thammasat University (Rangsit Campus), Khlong Nueng, Khlong Luang, Pathum Thani 12121, Thailand

## Abstract

This study aims to investigate the renoprotective effect of recombinant human erythropoietin (rhEPO) treatment could preserve tubular epithelial cell regeneration and ameliorate renal fibrosis by dual inhibition of stress-induced senescence and EMT in unilateral ureteric obstruction (UUO) mouse model. UUO or sham-operated mice were randomly assigned to receive rhEPO or vehicle treatment and were sacrificed on days 3, 7, and 14. Kidney specimens were fixed for histopathological and immunohistochemical study. The expression of S100A4, TGF-**β**1, BMP-7, Smad2/3, Smad1/5/8, and p16^INK4a^ was determined by western blot and real-time RT-PCR. Vehicle treated UUO mice had increased tubular atrophy and interstitial fibrosis within 3 to 14 days. An increase in TGF-**β**1, Smad2/3, S100A4, and p16^INK4a^ expression and a decrease in BMP-7 and Smad1/5/8 expression were observed in the obstructed kidneys. p16^INK4a^ was positively correlated with TGF-**β**1/Smad2/3 and negatively correlated with BMP-7/Smad1/5/8 in UUO mice. rhEPO treatment significantly suppressed the upregulation of TGF-**β**, Smad2/3, S100A4, and p16^INK4a^ and preserved the downregulation of BMP-7 and Smad1/5/8, resulting in markedly reduced TA/IF compared to the vehicle treated mice. The renoprotective effects of rhEPO could ameliorate renal TA/IF by modulating senescence and EMT which could be a part of therapeutic option in patients with chronic kidney disease.

## 1. Introduction

One striking feature observed during chronic kidney disease (CKD) is tubular atrophy and interstitial fibrosis (TA/IF). During chronic kidney injury, tubular epithelial cells (TEC) attempt to survive by activating proliferation, differentiation, stress induced senescence, and epithelial-to-mesenchymal transition (EMT) or apoptosis [1–3]. Proliferating cells can response by providing state of permanent cell cycle arrest termed cellular senescence [4, 5]. TGF-*β* is the potent cell cycle inhibitor, and Smad3 is a key mediator for TGF-*β* mediating the antiproliferative responses by inhibiting cell cycle progression from G_1_ to S phase [6]. The p16^INK4a^ is the major CDK inhibitors which prevent the passage through the G_1_ phase of the cell cycle by inhibiting CDK4 and CDK6 [7]. Furthermore, overexpression of Smad3 and TGF-*β* treatment induces p16^INK4a^ protein and mRNA expression in keratinocytes which contributes to growth arrest [8, 9]. Cellular senescence is characterized by not only the inability of cells to proliferation existing by lost the ability to divide and failed to grow, but also the maintenance of the cell viability and metabolic activity develop of the resistance to apoptosis and an altered pattern of gene expression by expression of cell cycle inhibitor genes [5]. Stress can be induced by extrinsic factors causing upregulation of cyclin dependent kinase inhibitor p16^INK4a^, resulting irreversible cell cycle arrest which could exhaust the repairing processes of TECs [10]. 

EMT is another important process affecting the population of interstitial fibroblasts which lose their epithelial phenotype and show the progressive development of a mesenchymal phenotype that leads to TEC damage and fibrosis in the kidney [11]. Many studies have demonstrated that TGF-*β* promotes renal fibrosis through EMT by activation of Smad2/3 [12–14] and was counteracted with BMP-7 by motivation of Smad1/5/8 to maintain the epithelial phenotype of TECs [15, 16]. The expression of the mesenchymal phenotype in TECs and fibroblasts using S100A4 as a marker has been observed in many kidney diseases [17, 18]. Consequently, it is important to develop a potential therapeutic target to prevent or reverse the process of stress-induced senescence and EMT to slow down the progression of CKD. 

Recombinant human erythropoietin (rhEPO) has been demonstrated to exert a renoprotective effect in addition to a hematopoietic effect in acute and chronic kidney injury [19–21]. It plays an important role in antiapoptosis, anti-inflammation, and antioxidation in many models of kidney diseases [19, 22]. Moreover, it has been reported that rhEPO treatment could slow progression of kidney injury through expansion of endothelial progenitor cells [23, 24]. Many clinical studies demonstrate that the early treatment of anemia in CKD patients with rhEPO can slow the progressive decline of renal function [25, 26]. 

In the present study, we investigated the advantage of rhEPO treatment in an unilateral ureteric obstruction (UUO) mouse model. We hypothesized that rhEPO treatment could have a renoprotective effect mediated through amelioration of stress-induced senescence and EMT. 

## 2. Materials and Methods

### 2.1. Animals Care and Experimental Model

The ethics committee in Thammasat University approved all experiments on animals. All animal experimentation was conducted in accord with the Thammasat Animal Experimental Unit Guidelines. Male ICR mice weighing 25–30 g were obtained from National Laboratory Animal Center (Mahidol University). All mice received tap water and a standard diet. Mice were anesthetized with pentobarbital sodium by intraperitoneal (IP) injection. The abdomen was shaved and soaked with betadine. A midline abdominal incision was made, and both kidneys and ureters were identified. The left ureter was dissected out and ligated with 4.0 silk at two points. The abdominal wound was sutured and the animals returned to the cages. Mice were divided into four experimental groups (total = 48). (1) Sham group (*n* = 6): mice were subjected to the surgical procedures except for the ureter ligation and received IP injection with vehicle. (2) Sham + EPO group (*n* = 6): these mice received IP injection with EPO dose 1,000 U/kg BW. (3) UUO group (*n* = 18): mice were subjected to the unilateral ureteral ligation and received IP injection with vehicle. (4) UUO + EPO group (*n* = 18): these mice were administered IP injection with EPO dose 1,000 U/kg BW. EPO and vehicle were administrated every other day from the day before operation to day 14. One-third of mice were sacrificed on day 3, one-third on day 7, and the other on day 14. Blood samples from all mice were obtained from hearts to measure hematocrit levels. The kidneys were harvested for various biochemical and morphological studies.

### 2.2. Hematocrit Measurement

At the end of each study group, hematocrit was measured for all mice. Blood sample was collected using heparin coated capillary tubes which were centrifuged and used to measure hematocrit with Micro Capillary reader (International Equipment Company, Inc.; Chattanooga, TN).

### 2.3. Renal Histology and Immunohistochemistry

Kidneys tissue was embedded in paraffin and 4 micrometer sections were stained with hematoxylin and eosin (H&E), periodic acid-Schiff (PAS), and Masson's trichrome. The percentage of histology changes, such as degree of glomerulosclerosis, tubular atrophy, and interstitial fibrosis, were evaluated under high power magnification (400x) in 5 to 10 consecutive fields, and mean percentages of histological change were then calculated.

Organs were fixed in 4% paraformaldehyde. Five-micrometer paraffin sections were dewaxed and rehydrated. Endogenous peroxidase was quenched with 3% H_2_O_2_ for 20 min and nonspecific binding blocked with 20% normal goat serum in phosphate-buffered saline. Sections were incubated at 4°C with primary antibodies against S100A4 (1 : 200; Abcam: Biomed Diagnostics (Thailand) Co. Ltd.), TGF-*β* (1 : 500; Santa Cruz Biotechnology), BMP-7 (1 : 200; Abcam: Biomed Diagnostics (Thailand) Co. Ltd.), Smad2/3 (1 : 200; Santa Cruz Biotechnology), and P16^INK4a^ (1 : 500; Santa Cruz Biotechnology), for at least 1 hr followed by secondary antibodies, and finally with diaminobenzidine substrate. Nuclei were counterstained with hematoxylin, and slides were dehydrated and mounted with permount.

### 2.4. Western Blotting

Protein samples were electrophoresed on 12% SDS-PAGE mini-gels and wet-transferred (Bio-Rad, ON, Canada) onto nitrocellulose membranes. Membranes were treated with blocking solution followed by an overnight incubation at 4°C with rabbit polyclonal antibody to TGF-*β* (Santa Cruz Biotechnology; 1 : 10,000 dilution), rabbit polyclonal antibody to Smad2/3 (Santa Cruz Biotechnology; 1 : 1,000 dilution), rabbit polyclonal antibody to BMP-7 (Abcam: Biomed Diagnostics (Thailand) Co. Ltd.); 1 : 500 dilution), rabbit polyclonal antibody to Smad1/5/8 (Santa Cruz Biotechnology; 1 : 1,000 dilution), mouse monoclonal antibody to P16^INK4a^ (Santa Cruz Biotechnology; 1 : 1000 dilution), and rabbit polyclonal antibody to S100A4 (Abcam: Biomed Diagnostics (Thailand) Co. Ltd.); 1 : 1,000 dilution) in 5% BSA-TTBS. The secondary antibody (PIERCE, IL, USA) was diluted to 1 : 5,000 in 5% BSA-TTBS and membranes treated for 1 hour at room temperature. Signals were visualized by chemiluminescent detection according to the manufacturers' instructions (PIERCE, IL, USA). Signals were quantified using GeneGnome Syngene Bio Imagine and GeneSnap image acquisition software (Syngene, MD, USA). 

### 2.5. Real-Time Polymerase Chain Reaction (RT-PCR)

Total RNA was extracted using the RNeasy mini kit (Qiagen, Chatworth, CA, USA) according to the manufacturers' instructions. High-quality RNA was eluted in 35 *μ*L RNase-free water. An aliquot of each RNA preparation was used to determine total RNA quality and concentration, measured at 260 nm (OD_260_). Pure RNA possessed an OD_260_/OD_280_ ratio of 1.6–1.9. Total RNA (0.25 *μ*g) was reverse-transcribed to cDNA by Taqman Reverse Transcriptase Reagent (Applied Biosystems, Roch Molecular Biochemical, NJ, USA) using random primers under the following cycling conditions: 25°C, 10 min; 48°C, 30 min; 95°C, 5 min. The mRNA levels of TGF-*β*, BMP7, Smad3, Smad8, P16^INK4a^, and hypoxanthine phosphoribosyltransferase (HPRT) were measured using an ABI PRISM 7700 Sequence Detection System (SDS version 1.6; PE Applied Biosystems). The primers and probes used are detailed in Table 1. All samples were subjected to RT-PCR along with the house keeping gene HPRT which was an internal standard and calibrator. The results were shown as fold increased or decreased over normal mice.

### 2.6. Statistical Analyses

Data were expressed as mean ± SD. Statistical analyses were carried out using the SPSS software (version 12.0). Statistically significant differences among groups were calculated by ANOVA Bonferroni and Mann-Whitney *U* tests using the least significant difference method. Statistical significance was defined as *P* < 0.05.

## 3. Results

### 3.1. rhEPO Treatment Did Not Affect Hematocrit Levels in UUO Mice

**Figure 1:**
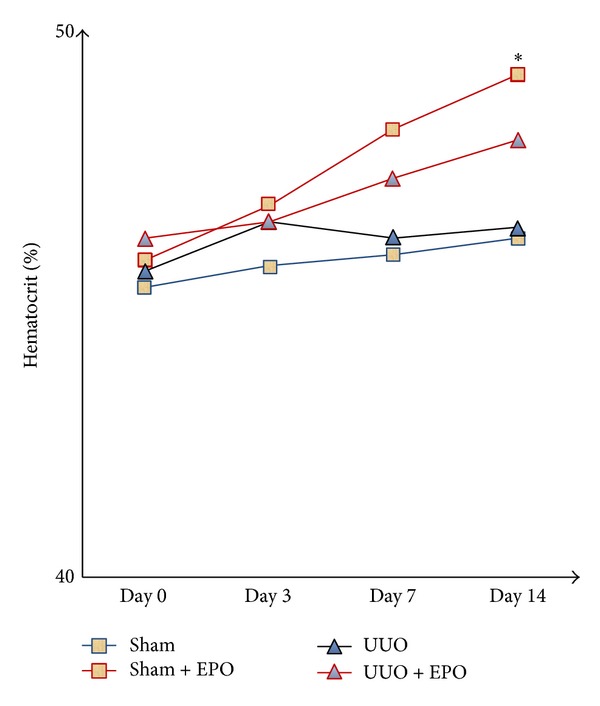
Representing the hematocrit values in sham and UUO mice treated with either vehicle or rhEPO. The hematocrit levels were not different over time between vehicle treated UUO and rhEPO treated UUO mice. *P < 0.05  versus  sham group.

 In sham control, hematocrit levels did not change during two weeks of experiment. Hematocrit levels in sham with rhEPO administration increased slightly at the end of the first week and then significantly increased only at the end of study (*P* < 0.05) when compared with vehicle treated sham (Figure 1). There were slightly increased hematocrit levels in rhEPO treated UUO mice during two weeks of experiment but no statistically significant difference when compared with vehicle treated UUO mice (Figure 1).

### 3.2. rhEPO Treatment Protected Against Renal Fibrosis in Mice UUO Model

**Figure 2:**
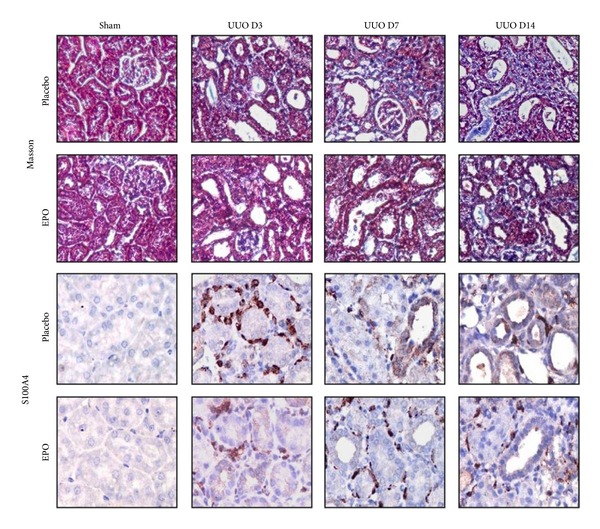
Masson's trichrome staining for assessing interstitial fibrosis in UUO mice. In sham kidney, no fibrosis labeling was seen. The obstructed kidneys show progressive interstitial fibrosis at days 3, 7, and 14 which was apparently ameliorated by rhEPO treatment. For immunohistochemistry, no S100A4 staining was detected in the sham kidneys. In UUO advanced increased S100A4 staining was seen on cells in interstitial area that displays the outline of lymphocytes and some TEC within the fibrosing obstructed kidneys at days 3, 7, and 14. However, decreased of S100A4 expression was observed in UUO mice with rhEPO treatment. Original magnifications ×400.

From H&E and PAS staining, the obstructed kidneys exhibited a significant progressive TA over time compared with the sham (*P* < 0.05), which was significantly reduced with the rhEPO treatment (*P* < 0.05) (Table 2). Masson's trichrome stained kidney sections showed a significant progression increase of collagen deposition in interstitial area over time after UUO compared with the sham (*P* < 0.05), which was significantly suppressed with the rhEPO treatment (*P* < 0.05) when compared with the vehicle treatment (Table 2 and Figure 2).

In sham kidneys, S100A4 was not detected in any tubule or interstitial area (Figure 1). However, S100A4 staining was detected in some TECs and cells in interstitial areas that displayed the outline of lymphocytes within the fibrosing obstructed kidneys. Staining of S100A4 progressively increased over time after UUO compared with the sham (Figure 2) and was reduced in the rhEPO compared with the vehicle treatment (Figure 2). By western blot analysis, the S100A4 level also increased progressively over time in UUO compared with the sham kidneys (*P* < 0.05) (Figure 4) but rhEPO treated UUO mice showed significantly decreased S100A4 levels at all time points compared with the vehicle treatment (*P* < 0.05) (Figure 4).

### 3.3. rhEPO Treatment Attenuated Increased TGF-*β* but Preserved BMP-7 in UUO Kidneys

**Figure 3:**
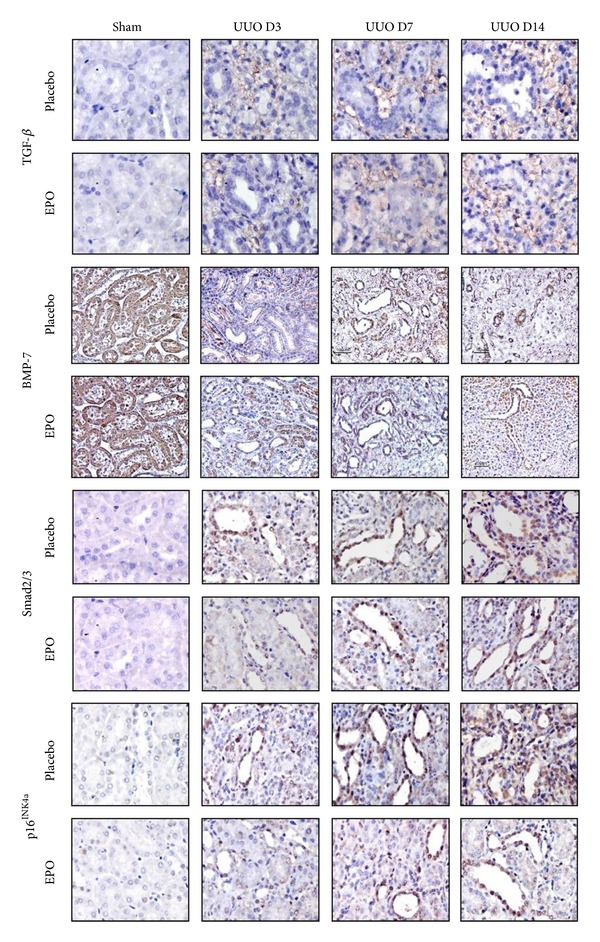
Representative photographs of kidney sections stained with TGF-*β*, BMP-7, Smad2/3, and p16^INK4a^ in UUO model. In sham kidneys, no or little TGF-*β* labelling was seen. Advanced increased TGF-*β* labelling was seen in the interstitium area in the obstructed kidneys compared with the sham at days 3, 7, and 14. In contrast, decrease of TGF-*β* staining was observed in UUO mice with rhEPO treatment. In contrast, BMP-7 was demonstrated in the cytoplasm of TEC in sham kidneys, whereas the labeling of BMP-7 was decreased in cytoplasm of TEC particularly in dilated and atrophic tubules of the placebo treated UUO kidneys since day 3 after UUO and progressive loss until day 14. rhEPO treatment in mice with UUO demonstrated the significantly preserved cytoplasm staining intensity of BMP-7 in the obstructed kidneys. Moreover, no Smad2/3 and p16^INK4a^ staining was seen in TEC in sham kidneys. Smad2/3 and p16^INK4a^ are detected at the nucleus of TEC with weak cytoplasm staining particularly in dilated and atrophic tubules of the UUO kidneys since days 3, 7, and 14. In contrast, rhEPO treatment in mice with UUO demonstrated the significantly attenuated nucleus and cytoplasm staining intensity of Smad2/3 and p16^INK4a^ in the obstructed kidneys. Original magnifications ×400.

**Figure 4:**
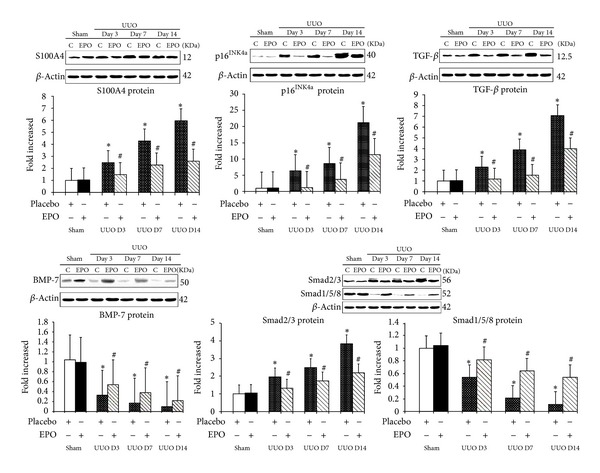
Western blot analyses depicting S100A4, TGF-*β*, BMP-7, Smad2/3, Smad1/5/8, and p16^INK4a^ expression *in vivo* after rhEPO treatment in UUO model. S100A4, TGF-*β*, Smad2/3, and p16^INK4a^ expression were significantly higher in UUO mice on days 3, 7, and 14 (*P* < 0.05) than in sham or sham + rhEPO. Treatment with rhEPO resulted in a decrease in S100A4, TGF-*β*, Smad2/3, and p16^INK4a^ expression (*P* < 0.05). In contrast, BMP-7, and Smad1/5/8 expression were significantly lower in UUO mice on days 3, 7, and 14 (*P* < 0.05) than in sham or sham + rhEPO. Treatment with rhEPO resulted in preserved BMP-7 and Smad1/5/8 expression (*P* < 0.05). *n* = 6 in each group. Each bar represents the mean ± SD. *P < 0.05  versus  sham group; ^#^ *P* < 0.05  versus  UUO group.

**Figure 5:**
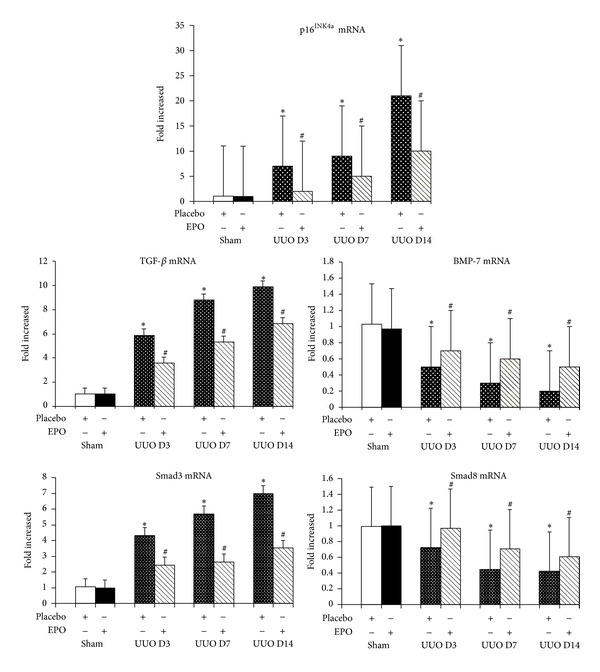
Real-time RT-PCR for TGF-*β*, BMP-7, Smad3, Smad8, and p16^INK4a^ mRNA expression *in vivo* after rhEPO treatment in UUO model. TGF-*β*, Smad3, and p16^INK4a^ mRNA expression show markedly progressive upregulation in UUO mice on days 3, 7, and 14 (*P* < 0.05) than in sham or sham + rhEPO. Treatment with rhEPO significantly downregulation of TGF-*β*, Smad3, and p16^INK4a^ mRNA expression compared with placebo treatment (*P* < 0.05). On the other hand, BMP-7 and Smad8 mRNA expression were significantly lower in UUO mice on days 3, 7, and 14 (*P* < 0.05) than in sham or sham + rhEPO. Treatment with rhEPO showed significantly the sluggish downregulation of BMP-7 and Smad8 expression (*P* < 0.05). *n* = 6 in each group. Each bar represents the mean ± SD. *P < 0.05  versus  sham group; ^#^ *P* < 0.05  versus  UUO group.

By immunohistochemistry, TGF-*β* revealed no labeling in sham kidneys but was strongly detected with increasing intensity in interstitial areas of vehicle treated obstructed kidneys over time after UUO (Figure 3). Western blot analysis demonstrated the progressively increased TGF-*β* in the obstructed kidneys compared with the sham kidneys (Figure 4). In contrast, UUO mice treated with rhEPO had decreased TGF-*β* staining over time (Figure 3) similar to western blot analysis revealing the significantly decreased TGF-*β* protein when compared with the vehicle treatment (*P* < 0.05) (Figure 4). Similarly, upregulation of TGF-*β* mRNA expression was demonstrated in the obstructed kidneys by RT-PCR (Figure 5) which significant downregulation in the rhEPO treated UUO mice.

In the sham kidneys, staining of BMP-7 was demonstrated in the cytoplasm of TECs, whereas in vehicle treated UUO kidneys there was a progressive loss of BMP-7 staining, particularly in dilated and atrophic tubules (Figure 3), which preserved staining in rhEPO treated UUO mice (Figure 3). Western blot analysis demonstrated significantly decreased BMP-7 in the obstructed kidneys compared with the sham (*P* < 0.05) (Figure 4), but significantly preserved BMP-7 in the rhEPO compared with the vehicle treated UUO mice (*P* < 0.05) (Figure 4). Moreover, a significant downregulation of BMP-7 mRNA expression was observed during the obstructive process (*P* < 0.05) (Figure 5). In contrast, the downregulation of BMP-7 mRNA expression in the UUO mice with the rhEPO was significantly slowed down compared with the vehicle treatment (*P* < 0.05) (Figure 5).

### 3.4. rhEPO Treatment Attenuated Increased Expression of Profibrosis Smad2/3 and the Decreased Expression of Antifibrosis Smad1/5/8 in UUO Kidneys

In sham kidneys, there was no Smad2/3 staining in the nucleus of TECs, whereas the staining of Smad2/3 was prominent in nucleus and some parts of cytoplasm of TECs particularly in dilated and atrophic tubules of the obstructed kidneys (Figure 3). In contrast, rhEPO treatment in mice with UUO demonstrated the significantly reduced nucleus staining intensity of Smad2/3 in the obstructed kidneys (Figure 3). Western blot analysis demonstrated the progressively increased Smad2/3 and decreased Smad1/5/8 protein in the obstructed kidneys compared with the sham (Figure 4). In contrast, rhEPO treated UUO mice showed significantly inhibited the rising of the Smad2/3 and maintained the declining of Smad1/5/8 compared with the vehicle treatment (*P* < 0.05) (Figure 4). Upregulation of Smad3 and downregulation of Smad8 mRNA expression were significantly changed during the obstructive processes (*P* < 0.05) (Figure 5). On the other hand, treatment with rhEPO in UUO mice significantly suppressed the upregulation of Smad3 mRNA and defended the downregulation of Smad8 mRNA expression compared with vehicle treated UUO mice (*P* < 0.05) (Figure 5).

### 3.5. rhEPO Treatment Inhibited Increased Expression of P16^INK4a^ in UUO Kidneys

In the sham kidneys, P16^INK4a^ staining was not detected in the nucleus of TECs, whereas P16^INK4a^ staining was prominent in the nucleus and some cytoplasm of TECs particularly in dilated and atrophic tubules of the UUO kidneys (Figure 3). rhEPO treated UUO mice showed significantly attenuated staining intensity of P16^INK4a^ in the nucleus and/or cytoplasm of TECs in the obstructed kidneys (Figure 3). Western blot and RT-PCR analyses also confirmed markedly increased P16^INK4a^ protein and mRNA expression in the UUO kidneys compared with the sham kidneys (*P* < 0.05) (Figures 4 and 5). In contrast, treatment with rhEPO in UUO mice showed significantly decreased P16^INK4a^ protein and mRNA expression compared with vehicle treatment (*P* < 0.05) (Figures 4 and 5).

### 3.6. Positive Correlation between TGF-*β*, Smad2/3, and p16^INK4a^ but Negative Correlation between BMP-7, Smad1/5/8, and p16^INK4a^ in UUO Kidneys

**Figure 6:**
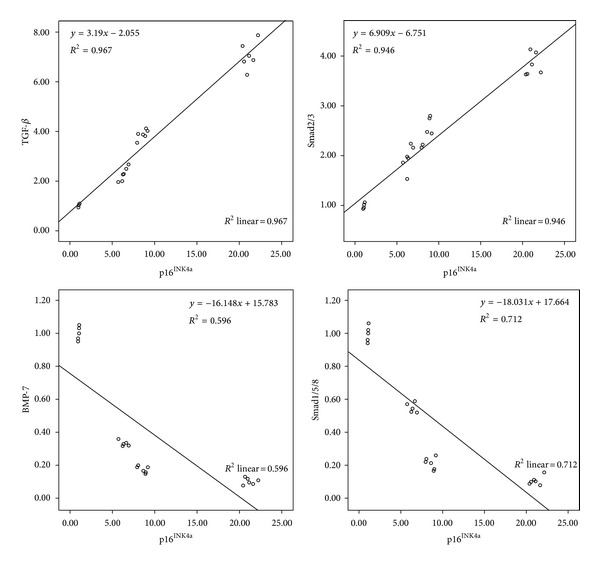
Representative regression analysis demonstrates the positive correlation between TGF-*β*, Smad2/3, and p16^INK4a^ protein in UUO mice (*P* < 0.001). In contrast, negative correlation between BMP-7, Smad1/5/8, and p16^INK4a^ protein in obstructed kidney was evaluated another time in the study (*P* < 0.001).

Obstructed kidneys had significantly increased TGF-*β*, Smad2/3, and p16^INK4a^ but decreased BMP-7, Smad1/5/8 protein, and mRNA. The p16^INK4a^ protein concentration was positively correlated with TGF-*β* (*R* ^2^ = 0.97, *P* < 0.001) and Smad2/3 (*R* ^2^ = 0.95, *P* < 0.001) (Figure 6) but was negatively correlated with BMP-7 (*R* ^2^ = 0.6, *P* < 0.001) and Smad1/5/8 (*R* ^2^ = 0.71, *P* < 0.001) (Figure 6).

## 4. Discussion

The present study demonstrates the renoprotective effects of rhEPO which ameliorate TA/IF in UUO mice and provides robust evidence for its mechanism of action. rhEPO reduced the effects of TGF-*β*, Smad2/3, p16^INK4a^, and S100A4 and enhanced the effects of BMP-7 and Smad1/5/8 which promote the regenerative process of TECs by inhibiting stress induced senescence and reduce fibrosis by attenuating EMT. 

### 4.1. Upregulation of TGF-*β*/Smad3 and Downregulation of BMP-7/Smad1/5/8 Expression in UUO Kidneys Associated with Senescence

 Ligating the ureter resulting in stress induced cellular senescence originates cell cycle arrest in obstructed kidney mice. The expression of p16^INK4a^ protein was present in most nuclei and some cytoplasm of TECs. Upregulation of p16^INK4a^ had a positive relationship with degree of TA/IF in obstructed kidney similar to renal aging and many kidney diseases [27–29]. TGF-*β* is known to be one of the most important cytokines for triggering stress induced senescence by increasing p16^INK4a^ [30] and Smad3, whereas Smad3 depletion reduced senescence [8, 9]. In contrast, BMP activated Erk1/2 and Smad1/5/8-Id1 pathways regulate p16^INK4a^ expression through phosphorylating BMP receptor IA inhibiting cellular senescence [31]. We showed a positive relationship between TGF-*β*, Smad3, and p16^INK4a^ and a negative relationship between BMP-7, Smad1/5/8, and p16^INK4a^. Thus, overexpression of TGF-*β*/Smad3 and underexpression of BMP-7/Smad1/5/8 during chronic kidney injury induce cellular senescence resulting in a widespread irreversible cell cycle arrest with limited TECs regeneration.

### 4.2. Upregulation of TGF-*β*/Smad2/3 and Downregulation of BMP-7/Smad1/5/8 Expression in UUO Kidneys Associated with EMT

During kidney development, BMP-7 plays an important role in promoting mesenchymal-to-epithelial transition (MET) and maintaining TEC epithelial phenotype [32] whereas TGF-*β* is a major cytokine that regulates EMT. UUO mice had increased TGF-*β*/Smad2/3 and decreased BMP-7/Smad1/5/8 expression that were directly associated with the degree of fibrosis. We showed increased staining and expression of S100A4 in the UUO mice, consistent with the development of EMT. Others have found that S100A4 is an early marker of fibroblast activity and that these fibroblasts are derived from epithelial cells and are a key pathophysiological step in the development of fibrosis [17, 33]. These findings suggest that, during the process of chronic kidney injury, TECs could be transformed into interstitial fibroblasts causing progression of renal fibrosis induced by overexpression of TGF-*β*/Smad2/3 and loss of BMP-7/Smad1/5/8 expression. 

### 4.3. Stress Induced Cellular Senescence and EMT Act Together to Limit Regenerative Capacity and Promote Fibrosis as the Chorus in Obstructed Kidney

In present study, we demonstrated that TECs exposed to cytokine majority by TGF-*β*/Smad2/3 and loss BMP-7/Smad1/5/8 which could coaccelerate the process of both senescence and EMT like the orchestra. We propose the hypothesis that the development of fibrosis during chronic kidney injury has two phases. In the early phase, stress induced cellular senescence is the major mechanism which promotes fibrosis. The changes in gene expression of any cell types are different during stress induced cellular senescence, suggesting that inflammatory response is cell type specific [5]. Because of senescent cells resistance to apoptosis, senescent TECs may accumulate over time as we found by p16^INK4a^ staining since day 3 after UUO and secrete a variety of proteins which stimulate the fibroblast come into the injured kidney. These fibroblasts display the highly activated phenotype characteristic of myofibroblasts by S100A4 expression but senescent TECs did not express it. Thus, fibroblasts from immune response play a major role to develop fibrosis in the early phase. In the later phase, EMT is the major mechanism which promotes fibrosis. We found the progressive staining of S100A4 in TECs at days 7 and 14 which correlated with fibrosis in the obstructed kidney. This finding means that TECs express more myofibroblasts phenotype and transformed to mesenchymal cells which invade into the interstitium area and turn to be the fibrosis. Hence, EMT by TECs plays a major process to develop fibrosis in the late phase.

### 4.4. rhEPO Treatment Demonstrated Renoprotective Effects in UUO Kidneys by Inhibiting Senescence and EMT

In the present study, TECs exposed to majority of proinflammatory TGF-*β*/Smad2/3 and loss of prosurvival BMP-7/Smad1/5/8 cytokines which could coaccelerate the process of both stress induced senescence by increasing p16^INK4a^ and EMT by increasing S100A4 during chronic inflammatory process. rhEPO suppressed the profibrotic TGF-*β*/Smad2/3 and maintained the anti-fibrotic BMP-7/Smads1/5/8 during the dynamic process of EMT by reducing mesenchymal marker S100A4 expression and fibrosis. Moreover, rhEPO treatment reduced senescent TECs in obstructed kidneys by inhibiting p16^INK4a^ expression thereby improving TECs regeneration. 

Chronic inflammatory processes frequently response during chronic kidney injury is characterised by increased the expression of proinflammatory and profibrotic cytokines such as TGF-*β*/Smad, tumor necrosis factor, and interleukin-6 activated fibrosis in the kidney [34–36]. Moreover, chronic kidney injury could accelerate stress induced senescence via increased p16^INK4a^ expression in the kidneys, as seen in chronic glomerulonephritis and chronic kidney allograft nephropathy that is associated with disease progression and limits regenerative capacity [29, 37, 38]. Activation of cells undergo senescence under chronic stress were stimulated through proinflammatory TGF-*β*/Smad3, tumor necrosis factor-*α* and Ras-MAPK pathways [39–41]. Many experimental studies have demonstrated that EPO administration protects TECs against apoptosis, chronic inflammation, and attenuated renal fibrosis through the interaction with the EPO receptor beyond anemia correction as seen in rats with 5/6 nephrectomy, full MHC-mismatched kidney transplantation, and UUO [42–45]. In rats with 5/6 nephrectomy, treatment with low dose long acting rHuEPO analogue protected the remnant kidneys by attenuated glomerulosclerosis and TA/IF with preserved renal function when compared with saline treatment which demonstrated the similar hematocrit levels. Low doses of rHuEPO treatment were safe and effective to protect the remnant kidneys and avoids adverse effects of high rHuEPO doses which cause an increase in hematocrit accompanied with changes in viscosity and activate thrombosis [42]. Recent study in rats with kidney transplantation and EPO treatment prevented chronic allograft dysfunction by ameliorated glomerulosclerosis, TA/IF, and inflammatory cell infiltration and preserved graft function. Correction of anemia in posttransplant anemic rats by blood transfusion did not prevent chronic allograft injury [43]. In UUO rat model, long term treatment with high dose EPO or carbamylated-erythropoietin (CEPO) significantly attenuated TA/IF when compared with saline. High dose EPO treatment significantly increased hematocrit than saline and CEPO treated rats but only CEPO treated rats had decreased TGF-*β*1 mRNA and fibrosis marker [44]. The present study, demonstrated that rhEPO treatment ameliorates TA/IF in UUO mice when compared with vehicle which display the comparable hematocrit levels. Thus, rhEPO treatment was associated with significantly reduced chronic kidney injury beyond the anemia correction, suggesting that improved anemia per se is unlikely to have a significant role in renal protection.

The renoprotective effect of EPO was mediated by activation of the antiapoptotic PI3K/Akt pathway in renal TECs [42, 43] and simultaneous decreases in TGF-*β*/Smad and tumor necrosis factor-*α* in the kidney [20, 21, 44–46]. Actually, EPO is secreted by fibroblast-like cells in the renal interstitium and acts on erythroid progenitor cells [47]. However, the interplay between EPO secreting cells, endothelial cells, mesangial cells, and TECs could facilitate endocrine and paracrine actions of EPO in the kidney. EPO receptors are present on many renal cells, including TECs [48]. Binding of a single EPO molecule to two adjacent EPO receptors on the membrane of target cells leads to homodimerization of the receptors and the triggering of different intracellular signaling pathways that might play an as yet unidentified role in cellular protection from stress. However, many studies proposed that the tissue protective effects of EPO are mediated through a tissue protective receptor that is distinct from the classical EPO receptor and is supposed to be a heteromeric receptor complex composed of EPO receptor and the ubiquitous *β*-common receptor (*β*cR, CD131) [49–52]. In the present study, we have shown that the major renoprotective effect of rhEPO was anti-inflammatory property. This effect not only decreases proinflammatory TGF-*β*/Smad2/3 but maintains anti-inflammatory BMP-7/Smad 1/5/8 cytokines also. Moreover, we found the evidence that rhEPO treatment can dual inhibit stress induced senescence and EMT, resulting simultaneously in supporting regenerative capacity and slowing the progression of fibrosis in the kidney.

## 5. Conclusions

In summary, we have provided robust evidence of the renoprotective effect of rhEPO which could ameliorate the progression of TA/IF in a mouse model of UUO as the source of cellular stress. rhEPO treatment protects against chronic renal injury by inhibiting stress induced senescence and EMT. These findings may explain partly the reduced rate of decline in renal function seen in CKD patients who have been treated with rhEPO for anemia and are consistent with the well-characterised antiapoptotic and anti-inflammatory properties of rhEPO in many models of chronic kidney injury. Studies on the role of rhEPO in reducing the rate of decline of renal function in different forms of CKD should be conducted.

## Figures and Tables

**Table 1:** Sequences of real-time PCR primers and probes.

Gene	Sequence
BMP-7	Forward 5′-TGGATGGGCAGAGCATCAA-3′ Reverse 5′-CTTGGAG CGATTCTGGCTG-3′ Probe 5′-FAM-ATTGGACGGCATGGACCCCAGA-TAMRA-3′

TGF-*β*1	Forward 5′-GGCTACCATGCCAACCAGCCTGGTGTACTCA-3′ Reverse 5′-CCGGGTTGTGTTGGTTGTAGA-3′ Probe 5′-FAM-CACACAGTACAGCAAGGTCCTTGCCCT-TAMRA-3′

Smad3	Forward 5′-GGGCCTACTGTCCAATGTCA-3′ Reverse 5′-CCCAATGTGTCGCCTTGTA-3′ Probe 5′-FAM-CCGGAATGCAGCCGTGGAAC-TAMRA-3′

Smad8	Forward 5′-CCTATCAACACTCAGACTTCCG-3′ Reverse 5′-GTGAAGCCGTCTATGAGCAC -3′ Probe 5′-FAM-ACTTTCCAGGCGTCCTCGCG-TAMRA-3′

P16^INK4a^	Forward 5′-GGACACCTTGAAGGAGGAGAAAG-3′ Reverse 5′-TTCTCCAACTCCTGGATGATGA-3′ Probe 5′-FAM-CCTTCAAGGCTTGGTTTCTCGTCAGACA-TAMRA-3′

HPRT	Forward 5′-TGACACTGGTAAAACAATGCAAACT-3′ Reverse 5′-AACAAAGTCTGGCCTGTATCCAA-3′ Probe 5′-FAM-TTCACCAGCAAGCTTGCAACCTTAACC-TAMRA-3′

**Table 2:** The percentage of histopathology changes, such as degree of glomerulosclerosis, tubular atrophy, and interstitial fibrosis in UUO mice.

	Sham		UUO					
	Day 0		Day 3		Day 7		Day 14	
	Placebo	EPO	Placebo	EPO	Placebo	EPO	Placebo	EPO
Glomerular sclerosis (%)	0.2 ± 0.4	0.2 ± 0.5	0.4 ± 0.5	0.3 ± 0.5	0.6 ± 0.5	0.5 ± 0.6	1.6 ± 0.5	1.5 ± 0.5
Tubular atrophy (%)	0.6 ± 0.5	0.4 ± 0.4	9 ± 2*	3 ± 1^#^	17 ± 4*	7 ± 3^#^	46 ± 2*	18 ± 3^#^
Interstitial fibrosis (%)	0.6 ± 0.5	0.4 ± 0.5	8 ± 3*	4 ± 2^#^	21 ± 4*	9 ± 2^#^	49 ± 4*	25 ± 4^#^

Values are means ± SD.

Significant difference *P < 0.05 compared to sham group; ^#^ *P* < 0.05 compared to UUO group by Mann-Whitney *U* test.
