# Corrigendum to “Dual Inhibiting Senescence and Epithelial-to-Mesenchymal Transition by Erythropoietin Preserve Tubular Epithelial Cell Regeneration and Ameliorate Renal Fibrosis in Unilateral Ureteral Obstruction”

**DOI:** 10.1155/2017/1357109

**Published:** 2017-03-19

**Authors:** Adis Tasanarong, Supranee Kongkham, Sookkasem Khositseth

**Affiliations:** ^1^Nephrology Unit, Department of Medicine, Faculty of Medicine, Thammasat University (Rangsit Campus), Khlong Nueng, Khlong Luang, Pathum Thani 12121, Thailand; ^2^Division of Biochemistry, Department of Preclinical Sciences, Faculty of Medicine, Thammasat University (Rangsit Campus), Khlong Nueng, Khlong Luang, Pathum Thani 12121, Thailand; ^3^Department of Paediatric, Faculty of Medicine, Thammasat University (Rangsit Campus), Khlong Nueng, Khlong Luang, Pathum Thani 12121, Thailand

In the article titled “Dual Inhibiting Senescence and Epithelial-to-Mesenchymal Transition by Erythropoietin Preserve Tubular Epithelial Cell Regeneration and Ameliorate Renal Fibrosis in Unilateral Ureteral Obstruction” [1], there was an error in Figure 3, where the picture for EPO/Sham in the Smad2/3 was incorrect.

 The correct EPO/Sham picture is shown in Figure 3.

## Figures and Tables

**Figure 3:**
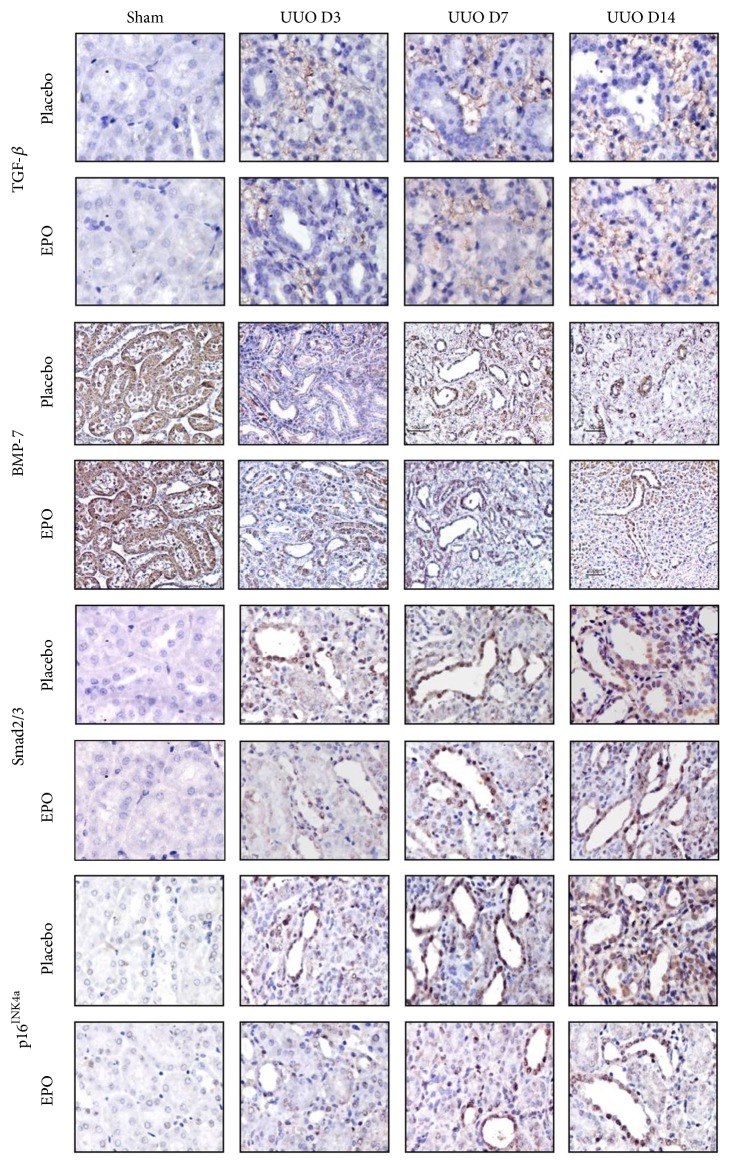
Representative photographs of kidney sections stained with TGF-*β*, BMP-7, Smad2/3, and p16^INK4a^ in UUO model. In sham kidneys, no or little TGF-*β* labelling was seen. Advance increased TGF-*β* labelling was seen in the interstitium area in the obstructed kidneys compared with the sham at days 3, 7, and 14. In contrast, decreased of TGF-*β* staining was observed in UUO mice with rhEPO treatment. In contrast, BMP-7 was demonstrated in the cytoplasm of TEC in sham kidneys, whereas the labeling of BMP-7 was decreased in cytoplasm of TEC particularly in dilated and atrophic tubules of the placebo treated UUO kidneys since day 3 after UUO and progressively loss until day 14. rhEPO treatment in mice with UUO demonstrated the significantly preserved the cytoplasm staining intensity of BMP-7 in the obstructed kidneys. Moreover, no Smad2/3 and p16^INK4a^ staining was seen in TEC in sham kidneys. Smad2/3 and p16^INK4a^ are detected at the nucleus of TEC with weak cytoplasm staining particularly in dilated and atrophic tubules of the UUO kidneys since days 3, 7, and 14. In contrast, rhEPO treatment in mice with UUO demonstrated the significantly attenuated nucleus and cytoplasm staining intensity of Smad2/3 and p16^INK4a^ in the obstructed kidneys. Original magnifications ×400.
